# Modulation of Adipocyte Differentiation and Proadipogenic Gene Expression by Sulforaphane, Genistein, and Docosahexaenoic Acid as a First Step to Counteract Obesity

**DOI:** 10.1155/2018/1617202

**Published:** 2018-02-07

**Authors:** Veronica Valli, Katharina Heilmann, Francesca Danesi, Alessandra Bordoni, Clarissa Gerhäuser

**Affiliations:** ^1^Department of Agri-Food Science and Technology (DISTAL), University of Bologna, Piazza Goidanich 60, 47521 Cesena, Italy; ^2^German Cancer Research Center (DKFZ), Division of Epigenomics and Cancer Risk Factors, Im Neuenheimer Feld 280, 69121 Heidelberg, Germany

## Abstract

Obesity is characterized by excess body fat accumulation due to an increase in the size and number of differentiated mature adipocytes. Adipocyte differentiation is regulated by genetic and environmental factors, and its inhibition could represent a strategy for obesity prevention and treatment. The current study was designed with two aims: (i) to evaluate the changes in the expression of adipogenic markers (C/EBP*α*, PPAR*γ* variant 1 and variant 2, and GLUT4) in 3T3-L1 murine preadipocytes at four stages of the differentiation process and (ii) to compare the effectiveness of sulforaphane, genistein, and docosahexaenoic acid in reducing lipid accumulation and modulating C/EBP*α*, PPAR*γ*1, PPAR*γ*2, and GLUT4 mRNA expression in mature adipocytes. All bioactive compounds were shown to suppress adipocyte differentiation, although with different effectiveness. These results set the stage for further studies considering natural food constituents as important agents in preventing or treating obesity.

## 1. Introduction

Obesity is the main dysfunction of adipose tissue and is associated with premature death and the development of chronic diseases such as cardiovascular diseases (CVD), type 2 diabetes, hypertension, and certain cancers [1]. In particular, a chronic inflammation in the absence of overt infection or autoimmune process is a puzzling phenomenon linked to obesity [2].

Environment, lifestyle, and genetic susceptibility certainly contribute to the increased risk of obesity, one of the easiest to be recognized and the most difficult to treat medical conditions [3]. Antiobesity drugs lack physiology specificity and have side effects [4].

Obesity is characterized by an excess accumulation of white adipose mass, resulting from both the increase in adipocyte cell size and the development of mature cells from undifferentiated precursors. Particularly, de novo generation of fat cells plays a key role in the development of obesity.

Discovering compounds able to regulate the size, number, and function of adipocytes and understanding their mechanisms of action could greatly contribute to obesity prevention and treatment. In this light, natural compounds represent a potential novel strategy, already exploited for preventing other metabolic disorders [5]. Bioactive compounds have been shown to exert specific effects on the biochemical and metabolic functions of adipocytes [6–8], in particular inhibition of preadipocyte differentiation, lipolysis stimulation, and induction of apoptosis of existing adipocytes [9], therefore contributing to a possible decrease in adipose tissue mass [10].

The aim of the current study was to compare the antiadipogenic effect of three bioactive compounds, namely, docosahexaenoic acid (DHA), genistein (GEN), and sulforaphane (SFN). DHA (C22:6 n-3) is an n-3 polyunsaturated fatty acid (PUFA) abundant in fatty fish. It is considered effective in the prevention of many chronic diseases, mainly CVD [11]. GEN (4,5,7-trihydroxyisoflavone), the most abundant isoflavone found in soybeans, has received particular attention for its structural similarity to estrogen and its high affinity to the estrogen receptor. It is a well-known antioxidant, chemopreventive, and anti-inflammatory agent [12, 13]. SFN, an isothiocyanate compound, is a constituent of cruciferous vegetables such as broccoli sprouts, Brussels sprouts, and cabbage. SFN is known to have antioxidant, immunomodulatory, anticancer, and antidiabetic properties [14, 15].

In some previous earlier studies [16–19], all tested bioactive compounds have been shown to be antiadipogenic in the 3T3-L1 cell line. Notwithstanding, to our knowledge, their effectiveness has never been compared in the same experimental conditions. Although *in vitro* studies always need confirmation *in vivo*, the selection of the most active bioactive could be useful to formulate functional foods contributing to the development of new strategies to prevent obesity.

3T3-L1 cells constitute the most frequently used preadipocyte model, sharing many properties with normal adipocytes [20]. Their differentiation into mature adipocytes involves the exposure of a confluent, quiescent population of cells to a variety of effectors that activate a complex cascade of genes [21].

It is well documented that adipogenesis is finely controlled by key transcription factors such as peroxisome proliferator-activated receptor-*γ* (PPAR*γ*) and CCAAT-enhancer-binding protein-*α* (C/EBP*α*). PPAR*γ* and C/EBP*α* regulate the expression of various genes involved in lipogenesis, lipolysis, and insulin sensitivity, such as the one encoding for glucose transporter type 4 (GLUT4) [22, 23]. In the first part of the study, changes in the expression of PPAR*γ*, C/EBP*α*, and GLUT4 genes were evaluated in the murine 3T3-L1 cell line at various stages of the differentiation process.

In the second part of the study, preadipocytes were supplemented during and after differentiation with DHA, GEN, and SFN, and both lipid accumulation and the mRNA expression of PPAR*γ*, C/EBP*α*, and GLUT4 were evaluated to evidence and compare their potential inhibitory activity on adipogenesis.

## 2. Materials and Methods

Dulbecco's modified Eagle's medium (DMEM)/F12 GlutaMAX I was purchased from Invitrogen (Darmstadt, Germany), donor bovine serum (DBS) was from Gibco Life Technologies (Darmstadt, Germany), fetal bovine serum (FBS GOLD) was from PAA Laboratories (Pasching, Austria), and TRIzol Reagent was from Ambion, Life Technologies (Darmstadt, Germany). All other chemicals were purchased from Sigma-Aldrich (Schnelldorf, Germany) and were of the highest analytical grade.

### 2.1. Cell Culture and Differentiation

3T3-L1 mouse preadipocytes were obtained from the American Type Culture Collection (ATCC) and maintained at 37°C in a humidified atmosphere containing 95% air and 5% CO_2_; preadipocytes were subcultured every three days when 80% confluent or less into a new 175 cm^2^ flask. Cells were cultured in DMEM/F12 GlutaMAX I with the addition of D-glucose (3151 mg/L f.c.) (GM) containing 10% DBS and 1% penicillin/streptomycin (PS). Cells were seeded in 12-well plates or a 25 cm^2^ flask at a concentration of 50,000 cells/mL. Three days after seeding, cells were stimulated to differentiate with GM supplemented with 10% FBS, 1% PS, insulin (10 *μ*g/mL), dexamethasone (1 *μ*M), isobutylmethylxanthine (0.2 mM), and rosiglitazone (10 *μ*M) (differentiation medium). After further 3 days (differentiation), cells were maintained in GM with FBS, PS, and insulin (postdifferentiation medium) for another 5 days (postdifferentiation) when approximately 90% of the cells displayed the characteristic lipid-filled adipocyte phenotype.

### 2.2. Bioactive Supplementation

DHA, GEN, and SFN were added to the differentiation and postdifferentiation medium at three different final concentrations (10, 25, or 50 *μ*M). The SRB assay was performed in preliminary experiments, evidencing no cytotoxicity for any of the tested concentrations of each bioactive.

The treatment with bioactives began three days after seeding and lasted until the end of postdifferentiation (eleven days from seeding). All bioactive compounds were purchased from Sigma-Aldrich (Schnelldorf, Germany). Each compound was dissolved in dimethyl sulfoxide (DMSO). Unsupplemented control cells (CTR) received a corresponding amount of DMSO (<0.5% final concentration). The medium was changed every two days during postdifferentiation.

### 2.3. Lipid Staining

The effect of the bioactive compounds on adipogenesis was evaluated morphologically by staining accumulated lipids with Oil Red O [24] as previously described [25]. Briefly, cells were fixed with 4% formalin solution in phosphate-buffered saline (PBS) for two hours, washed with water, rinsed with isopropanol 60%, and stained with Oil Red O for 30 minutes at room temperature. After washing with distilled water for 3 times, the lipid droplets were quantified by dissolving Oil Red O in isopropanol 100% and measuring the optical density at 500 nm.

The lowest bioactive concentrations able to influence lipid accumulation (10 *μ*M GEN, 10 *μ*M SFN, 25 *μ*M DHA) were then used in gene expression experiments.

### 2.4. Gene Expression Analysis

Unsupplemented, control cells were collected at four different steps of the differentiation protocol: one day after seeding (T1); three days after seeding (postconfluent cells), before the beginning of differentiation (T2); six days after seeding (end of the differentiation), before the addition of the postdifferentiation medium (T3); and eleven days after seeding, at the end of postdifferentiation (T4). Cells were collected at the different time points, and total RNA was extracted as described below.

In experiments evaluating bioactives' effect, 10 *μ*M GEN, 10 *μ*M SFN, or 25 *μ*M DHA was added to the differentiation and postdifferentiation media as described above. At the end of the postdifferentiation period (T4), cells were collected, and total RNA was extracted with TRIzol Reagent following the manufacturer's protocol. Contaminating DNA was eliminated by DNase treatment (DNA-free Kit from Ambion, Life Technologies, Darmstadt, Germany). RNA quantity and quality, respectively, were assessed by spectrophotometric analyses at 260/230 nm using a NanoDrop ND-2000 spectrophotometer (Thermo Fisher Scientific, Wilmington, DE, USA) and by the microfluidics-based Bioanalyzer platform with an RNA Nano 6000 Chip (Agilent Technology, Waldbronn, Germany).

cDNA was synthesized from 0.5 *μ*g or 1 *μ*g of DNase-treated total RNA using SuperScript II reverse transcriptase (Invitrogen, Darmstadt, Germany) according to the manufacturer's instructions. Quantitative real-time PCR (qPCR) was performed using the Universal ProbeLibrary system (Roche, Mannheim, Germany) on a Roche LightCycler 480 real-time PCR system (Roche, Mannheim, Germany). The cycling program for analysis was 15 min at 95°C followed by 45 cycles of 10 s at 95°C, 20 s at 55°C, and 10 s at 72°C with the primer pairs and the respective monocolor hydrolysis probes indicated in Table 1. The expression levels of target mRNAs were normalized to three reference genes: *β*-actin (ACTB), hypoxanthine phosphoribosyltransferase 1 (HPRT1), and TATA-box-binding protein (TBP).

### 2.5. Statistical Analysis

Gene expression data were analyzed using DataAssist software version 3.01 (Applied Biosystems, Foster City, CA, USA) and expressed as the mean fold change in relative expression compared with the untreated control cells, which were normalized to one. Average fold change and standard deviation (SD) were obtained from three biological replicate samples per condition.

All data were analyzed by one-way ANOVA, followed by Dunnett's or Tukey's tests. Statistical analysis of the data was performed using the GraphPad Prism 5 software (San Diego, CA, USA).

## 3. Results

### 3.1. Characterization of Preadipocyte Differentiation

**Figure 1:**
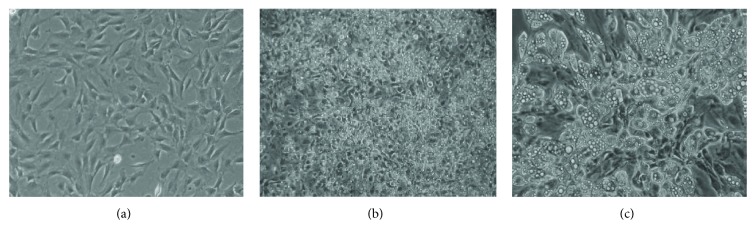
Morphological changes among (a) preadipocytes three days after seeding (T2), (b) adipocytes at the end of the differentiation (T3), and (c) adipocytes at the end of postdifferentiation (T4). Images showing different cell morphologies were captured at the different steps using a Leica DM IL microscope (Wetzlar, Germany), with 10x magnification.

During differentiation (T1–T4), preadipocytes acquired the characteristics of mature adipocytes. At three days after seeding (T2), nondifferentiated cells showed typical fibroblastoid morphology, while at the end of the differentiation process (T4), cells had abundant intracytoplasmic lipid accumulation, showing typical white adipocyte morphology (Figure 1).

To characterize the differentiation process, PPAR*γ*1, PPAR*γ*2, C/EBP*α*, and GLUT4 gene expression was evaluated at four different stages of adipocyte differentiation: one day after seeding (T1), three days after seeding (T2), at the end of the differentiation (T3), and at the end of postdifferentiation (T4).

**Figure 2:**
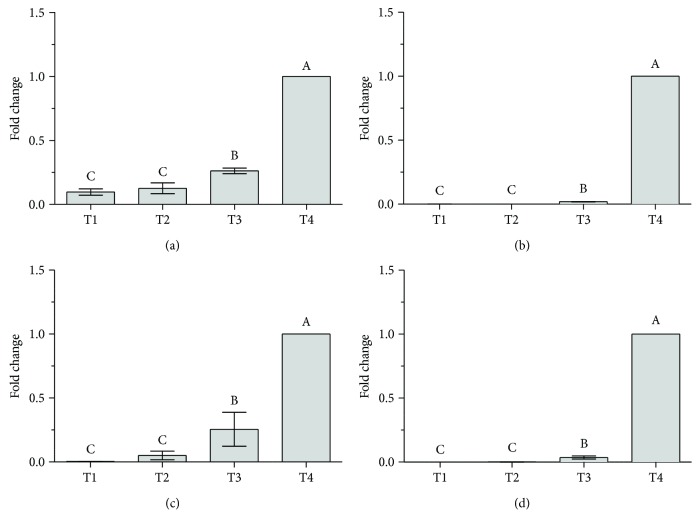
(a) PPAR*γ*1, (b) PPAR*γ*2, (c) C/EBP*α*, and (d) GLUT4 mRNA expression at 4 different stages of adipocyte differentiation. T1: one day after seeding; T2: three days after seeding, before the beginning of differentiation; T3: at the end of the differentiation, before the addition of the postdifferentiation medium; T4: at the end of postdifferentiation (mature adipocytes). Data are expressed as the mean fold change relative to the mature cells (T4), normalized to one. Statistical analysis was by one-way ANOVA (*p* < 0.001 for all panels) followed by Tukey's HSD test. The expression is significantly different between groups marked with different letters (at least *p* < 0.05).

The expression of selected genes was very low and similar at T1 and T2, while it significantly increased at T3. For all analyzed genes, a prominent increase in mRNA levels was observed in mature adipocytes (T4) (Figure 2).

### 3.2. Effects of Bioactive Compound Supplementation

**Figure 3:**
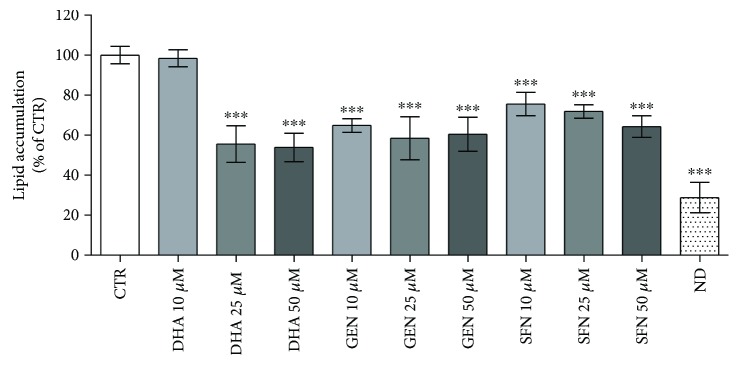
Lipid accumulation in supplemented and control cells. Data are expressed as a percentage relative to unsupplemented control cells (CTR), assigned as 100%. Statistical analysis was performed by one-way ANOVA (*p* < 0.001) followed by Dunnett's test: ^∗∗∗^*p* < 0.001 versus CTR. ND: nondifferentiated cells, before the beginning of the differentiation process.

The antiadipogenic potential of DHA, GEN, and SFN was first investigated evaluating their influence on lipid accumulation. Preadipocytes were supplemented with different concentrations (10, 25, and 50 *μ*M) of the test compounds during the differentiation and postdifferentiation periods, as described above, and lipid accumulation was detected by Oil Red O staining. All bioactive compounds markedly reduced lipid droplet formation compared to controls. GEN and SFN were effective at the lowest concentration used for supplementation (10 *μ*M), while a higher DHA concentration (25 *μ*M) was required to reduce lipid accumulation (Figure 3).

The lowest bioactive concentrations causing a significant decrease in lipid accumulation were used to verify the modification in the mRNA levels of the adipogenesis marker genes after differentiation.

**Figure 4:**
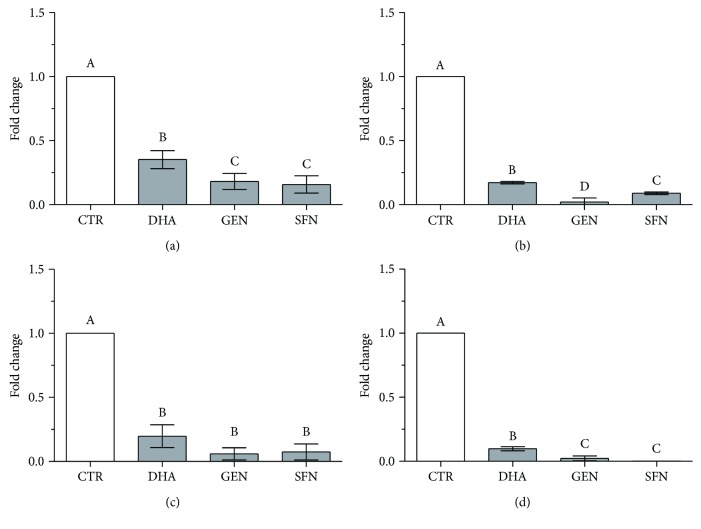
Modulatory effect of GEN, SFN, and DHA on (a) PPAR*γ*1, (b) PPAR*γ*2, (c) C/EBP*α*, and (d) GLUT4 mRNA expression. Data are expressed as the mean fold change relative to the unsupplemented control cells (CTR) at T4, normalized to one. Statistical analysis was by one-way ANOVA (*p* < 0.001 for all panels) followed by Tukey's HSD test. The expression is significantly different between groups marked with different letters (at least *p* < 0.05).

At T4, all bioactive compounds significantly reduced the transcript levels of PPAR*γ*1, PPAR*γ*2, C/EBP*α*, and GLUT4. The effect of GEN and SFN on PPAR*γ* and GLUT4 expression appeared stronger than the DHA effect did (Figure 4).

## 4. Discussion

Adipose tissue has an important function in the energy balance by regulating lipid metabolism, glucose homeostasis, and adipokine secretion. Thus, its dysfunction is critical in developing metabolic diseases [26]. Indeed, the incidence of metabolic syndrome, a combination of cardiometabolic risk determinants, is increasing worldwide largely as a consequence of the continued obesity epidemic [27].

In general, obesity is related to the extent of adipocyte differentiation, intracellular lipid accumulation, and lipolysis [17]. The process of adipocyte differentiation requires the activation of numerous transcription factors which are in charge of the coordinated induction and silencing of more than 2000 genes [28]. Several transcriptional regulators, including C/EBP and PPAR*γ*, play a pivotal role in this process.

The master regulator PPAR*γ* is both necessary and sufficient for adipogenesis [29, 30]. PPAR*γ* has two isoforms, the ubiquitary PPAR*γ*1 and the adipose tissue-specific PPAR*γ*2. Both isoforms are strongly induced during preadipocyte differentiation [25], and our data confirm that PPAR*γ*1 induction foreruns PPAR*γ*2 induction [31].

C/EBP*β* and C/EBP*δ* are overexpressed in the earlier phases of differentiation and have been shown to play a role in PPAR*γ* induction [30]. C/EBP*α* is involved in the maintenance of the terminally differentiated adipocyte phenotypes [28, 32, 33]. In agreement, we observed a lower C/EBP*α* expression at T1 and T2 than at T3 and T4.

In adipocytes, C/EBP*α* regulates the expression of the gene encoding for GLUT4, the major insulin-responsive glucose transporter in adipose tissue as well as in skeletal and cardiac muscles [34]. Accordingly, in the present study, GLUT4 expression paralleled C/EBP*α* expression.

Overall, our results confirm that differentiation of 3T3-L1 cells includes distinguishable multiple stages [35, 36].

Our results evidence that all tested bioactives efficiently block adipocyte differentiation. At T4, the expression of all the tested genes was significantly lower in supplemented cells than in unsupplemented ones and comparable to the expression level observed in unsupplemented cells at the first stages of differentiation.

The antiadipogenic effect of DHA, GEN, and SFN has been already reported in previous earlier studies [16–19]. Our study is not simply a confirmation that the tested bioactives act mainly through modification of the adipocyte life cycle [8], but mainly a representation of the first study comparing the effectiveness of DHA, GEN, and SFN in the same experimental conditions. Although GEN and SFN appeared effective at lower concentrations than DHA did, it is worth noting that *in vivo* the latter is absorbed and delivered to peripheral cells in its parent form. GEN and SFN are extensively metabolized, and they are detectable at very low concentrations in the bloodstream [37–39]. On the contrary, the DHA concentration used in this study for cell supplementation is easily reachable *in vivo* in the human plasma [40–42].

## 5. Conclusions

Our results represent an additional step in the evaluation of the antiadipogenic effects of three natural bioactive molecules, DHA, GEN, and SFN. Although *in vitro* all tested bioactive compounds appeared to be putative contributors to the prevention and treatment of obesity, their *in vivo* metabolism suggests that mainly DHA could potentially be used for the formulation of new functional food products devoted to a new dietetic natural strategy for overweight counteraction.

Further investigations are needed to verify whether the antiadipogenic properties evidenced *in vitro* do translate into *in vivo* efficacy in humans and to sort out the pathway(s) responsible for the beneficial effects. Moreover, the compounds here considered have been studied as discrete molecules and not as part of a food, ignoring both the matrix effect and the eventual synergistic or enhanced activities between the selected compounds and other food components or other bioactive molecules [43]. This issue also deserves future attention.

## Figures and Tables

**Table 1:** Primer sequences used in qPCR.

Gene	Forward primer	Reverse primer	Probe number
Target genes			
PPAR*γ*1	GAAAGACAACGGACAAATCACC	GGGGGTGATATGTTTGAACTTG	7
PPAR*γ*2	TGCTGTTATGGGTGAAACTCTG	CTGTGTCAACCATGGTAATTTCTT	2
C/EBP*α*	AAACAACGCAACGTGGAGA	GCGGTCATTGTCACTGGTC	67
GLUT4	GACGGACACTCCATCTGTTG	GCCACGATGGAGACATAGC	5
Reference genes			
ACTB	GTGGGAGAGCAAGGAAGAGA	CACTCTTGGCCCAGTCTACG	56
HPRT1	TCCTCCTCAGACCGCTTTT	CCTGGTTCATCATCGCTAATC	95
TBP	CGGTCGCGTCATTTTCTC	GGGTTATCTTCACACACCATGA	107
